# Periodontal Disease and Age-Related Macular Degeneration: A Meta-Analysis of 112,240 Participants

**DOI:** 10.1155/2020/4753645

**Published:** 2020-09-28

**Authors:** Xuewen Lv, Weiqi Li, Zhiyu Fang, Xiaofei Xue, Chunling Pan

**Affiliations:** ^1^School and Hospital of Stomatology, China Medical University, Shenyang, China; ^2^Liaoning Province Key Laboratory of Oral Diseases, Shenyang, China

## Abstract

**Objective:**

Epidemiological studies have shown a correlation between periodontal disease (PD) and age-related macular degeneration (AMD). However, the results have been inconsistent, and no relevant meta-analysis has been performed on this topic. Hence, we performed a meta-analysis to evaluate whether the two diseases are related. *Material and Methods*. The PubMed, Embase, Cochrane Library, and Web of Science databases were searched up to April 20, 2020, for related articles. Two authors independently conducted literature screening and data extraction and then used the Stata 15.1 software to calculate the relative risk (RRs) and 95% confidence intervals (CIs) to assess the association between PD and AMD.

**Results:**

A total of 5 observational studies involving 112,240 participants and 5,005 AMD patients were included. The results of meta-analysis using the random-effects model showed that the incidence of AMD in PD patients was 1.35 times that of non-PD patients; the difference was statistically significant (RR = 1.35, 95%CI = 1.07–1.70, *P* = 0.011). Sensitivity analysis showed that the results were stable.

**Conclusions:**

PD patients have a higher risk of AMD, but the causal relationship between PD and AMD has not been confirmed. Further research should be carried out to verify the exact relationship between the two.

## 1. Introduction

Age-related macular degeneration (AMD) is an eye disease characterized by central visual impairment and can cause severe irreversible vision loss. It is an important cause of blindness and central blindness in people over 55 years of age [1]. In developed countries, AMD is the first cause of blindness [2]. AMD has two main types of macular degeneration, that is, dry AMD (nonexudative AMD) and wet AMD (exudative AMD). The main risk factors for AMD include age, smoking, family history and genetic factors, diet and nutritional factors, obesity, and cardiovascular disease (CVD) [3]. The pathogenesis of AMD is still unclear, and its formation mechanism may be the result of complex multifactor interactions between metabolic, functional, genetic, and environmental factors [4]. The treatment methods currently used are mainly for wet AMD, and there is no effective treatment for dry AMD, which accounts for the majority of clinical cases. The future research direction of treatment may be to prevent and regulate risk factors, so in-depth research on the risk factors for AMD is important [5].

Periodontal disease (PD) is an inflammatory and destructive disease that destroys the periodontal support tissue (gingival, periodontal ligament, alveolar bone, and cementum), leading to the formation of periodontal pockets, loss of attachment, and alveolar bone resorption. As the lesion progresses, teeth loosen, gums recede, and eventually tooth loss occurs [6]. The systemic promoters of periodontal disease include genetics, sex hormones, smoking, related systemic diseases (such as diabetes and immune dysfunction), and mental stress [7]. Many studies have shown that periodontal disease has a two-way relationship with systemic health or diseases and is an important risk factor for cardiovascular and CVD, diabetes, and respiratory diseases [8–12].

Although the exact pathogenesis of AMD is still unclear, the theory of inflammation and immunology is gaining more and more attention, and the role of various inflammatory factors and immune factors has been confirmed by experiments and histopathological studies [13–15]. One possible mechanism for PD to affect systemic diseases is that periodontal bacteria and their metabolites (such as endotoxin) enter the systemic circulation, and when they reach other parts of the body, they activate monocytes/macrophages and produce a large number of inflammatory factors, which in turn causes inflammation in other organs [16, 17]. Given that PD and AMD share some common risk factors, and PD may cause AMD's immune response and inflammation through bacteria and their products, Klein et al. [18] reported that the history of periodontal disease was related to the increase in retinal pigment. Since then, some other relevant epidemiological studies had been published, but these studies had provided inconsistent results [18–22]. There is no relevant meta-analysis to quantitatively evaluate the connection between the two. The purpose of this study is to systematically review existing literature and use meta-analysis to analyze whether PD and AMD are independently related.

## 2. Materials and Methods

This study was based on the statement of the Preferred Reporting Items for Systematic Reviews and Meta-Analyses (PRISMA) [23].

### 2.1. Eligibility Criteria

Studies that met the following conditions can be included: (1) the research theme was to explore whether PD is related to AMD; (2) observational studies, including case-control studies, cross-sectional studies, and cohort studies; (3) studies that provided raw data, or relative risk (RRs), odds ratios (ORs), or hazard ratios (HRs), and their 95% CIs; (4) full-text articles were available and published in English, excluding letters to editors, meeting abstracts, case reports, and reviews; and (5) the study included a control group.

When different studies of the same population were included, we selected a longer follow-up period or extract data from more complete studies. The two authors (XWL and WQL) chose independently, and if there were differences, they resolved through discussion or consultation with the third author (CLP).

### 2.2. Search Strategy

A computerized search of the PubMed, Embase, Cochrane Library, and Web of Science databases was performed. We collected case-control studies, cross-sectional studies, and cohort studies of the relationship between PD and AMD; the search time limit was from the establishment of the database to April 20, 2020. In addition, the references included in the literature are retrospectively included to supplement the relevant literature. The following keywords and thematic terms were used: “Periodontal Diseases”, “Age-Related Macular Degeneration”, “Drusen”, “Geographic Atrophy”, “Retinal Pigment Epithelial Detachment”, “Choroidal Neovascularization”, and “Polypoidal choroidal vasculopathy”. In Supplementary Materials, we have given specific electronic search criteria for each of the above databases (Appendix 1).

### 2.3. Data Extraction

The two authors (XWL and WQL) independently used standardized tables for data extraction, and disputes were resolved through discussion or consultation with the third author (CLP). Contents included the first author, published year, sample, study period, study design, follow-up period, age, AMD patients/participants, PD ascertainment, AMD ascertainment, AMD/age status, adjusted RRs (including ORs/HRs), and 95% CIs, as well as adjustment factors and information required for quality assessment.

### 2.4. Quality Evaluation

Case-control studies and cohort studies used the Newcastle-Ottawa scale (NOS) for risk assessment of bias [24], and cross-sectional studies were evaluated by the bias risk evaluation criteria recommended by the Agency for Healthcare Research and Quality (AHRQ) [25]. The NOS scale includes a total of 8 items in three parts. The semiquantitative principle of the star system was used to evaluate the quality of the research. The full score is 9 stars; 0-3 stars, 4-6 stars, and 7-9 stars were defined as low, medium, and high quality. The standard recommended by AHRQ consists of 11 items, which were answered with “yes,” “no,” and “unclear,” respectively. If the answer of an item was “no” or “unclear,” the item's score was “0.” If the answer was “yes,” then the project score was “1”; 0-3 points, 4-7 points, and 8-11 points were defined as low, medium, and high quality. Disagreements were resolved through consensus between the two authors (XWL and WQL) or arbitration by the third author (CLP). When the study quality was defined as medium or high quality, the data in this study was included for meta-analysis.

### 2.5. Statistical Analysis

In observational studies, for small-probability events, OR can be considered approximately equal to RR [26]. For cohort studies that report HR because of survival data, HR is approximately RR [27]. Some studies only give the respective effect sizes and their 95% CIs for each severity of PD or AMD and did not give the total effect size and its 95% CI representing the correlation between PD and AMD. In order to observe whether PD and AMD are related in general, we used the fixed-effects model to combine the data of different degrees of disease in the same study, so as to obtain the total effect size and 95% CI of the study [28]. We converted the effect sizes and their 95% CIs into their logarithms and standard errors and then merged them. A chi-squared test was used to analyze the heterogeneity between the included studies. At the same time, *I*^2^ was used to quantitatively judge the heterogeneity, and the inspection level was set to *α* = 0.10. When *P* value < 0.1 or *I*^2^ > 50%, obvious heterogeneity was defined [29]. Usually, the random-effects model is used when there is obvious heterogeneity; otherwise, the fixed-effects model is used [29]. However, considering the obvious clinical heterogeneity caused by the different diagnostic criteria of PD and AMD and the methodological heterogeneity caused by the design and quality of studies, we decided to choose a random-effects model for statistical analysis. Subgroup analysis is based on study design, study areas, whether to adjust smoking, diabetes, hypertension, CVD, hepatitis B infection, study quality, and age status. Sensitivity analysis was to delete one study at a time and combined the remaining study data through a random-effects model to explore the degree of impact of the study on the total effect sizes and the robustness of the results. Since the number of studies was less than 10, no funnel plots were made. Begg's test and Egger's test were used to detect publication bias [30, 31]. All statistical analyses were completed by the two authors independently using Stata version 15.1 and R version 4.0.0, and the *P* values < 0.05 were considered statistically significant. Disagreements were resolved through discussion by the two authors (XWL and WQL) or consultation with the third author (CLP).

## 3. Results

### 3.1. Study Selection and Characteristics

**Figure 1:**
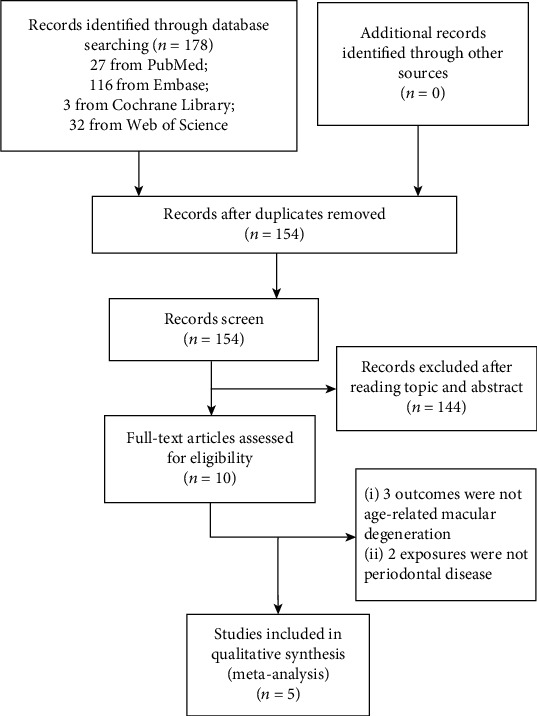
Search flow chart of the meta-analysis.

A total of 178 relevant literatures were obtained in the initial examination. After layer-by-layer screening, 5 studies were finally included [18–22], including 112,240 participants and 5,005 AMD patients. The search flow chart is shown in Figure 1. The included studies were published between 2008 and 2019 and included 4 cross-sectional studies and 1 retrospective cohort study. The characteristics of the studies are shown in Table 1. Five studies had adjusted the confounding factors, and Table 2 lists the detailed information. We evaluated the quality of all included studies. One item was classified as medium quality and four items as high quality (Tables 3 and 4).

### 3.2. Results of Meta-analysis

#### 3.2.1. Overall Estimation

**Figure 2:**
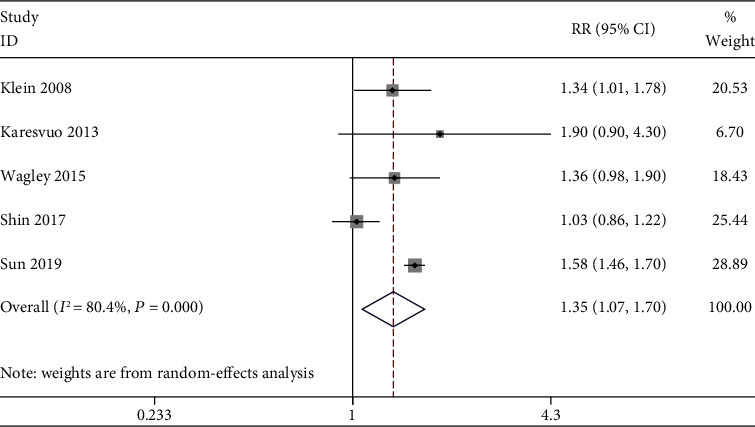
Forest plot of studies.

Meta-analysis results using the random-effects model showed that the incidence of AMD in PD patients was 1.35 times that of non-PD patients, and the difference was statistically significant (RR = 1.35, 95%CI = 1.07–1.70, *P* = 0.011). The heterogeneity test results found obvious heterogeneity (*I*^2^ = 80.4%, *P* for heterogeneity < 0.001). Detailed information is listed in Figure 2.

#### 3.2.2. Sensitivity Analysis

**Figure 3:**
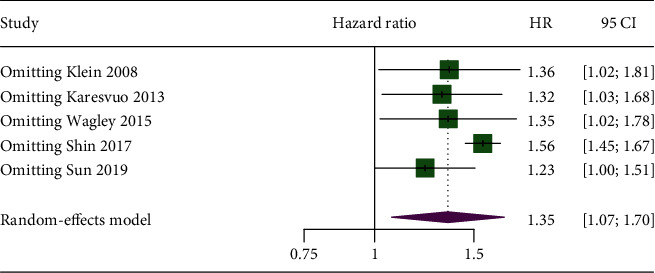
The results of the sensitivity analysis.

Figure 3 lists the results of the sensitivity analysis. After excluding the included studies one by one, the results showed no substantial changes, suggesting that the meta-analysis results were robust. After excluding Shin et al.'s study [21], the combined RR = 1.56 (95% CI: 1.45-1.67, *I*^2^ = 0.0%, heterogeneity for *P* = 0.550). After removing the cohort study by Sun et al. [22], the combined RR = 1.23 (95% CI: 1.00-1.51, *I*^2^ = 42.1%, heterogeneity for *P* = 0.159). The results did not change, and the heterogeneity decreased, indicating that these two studies contributed greatly to the statistical part of the overall meta-analysis heterogeneity.

#### 3.2.3. Subgroup Analysis and Metaregression

**Figure 4:**
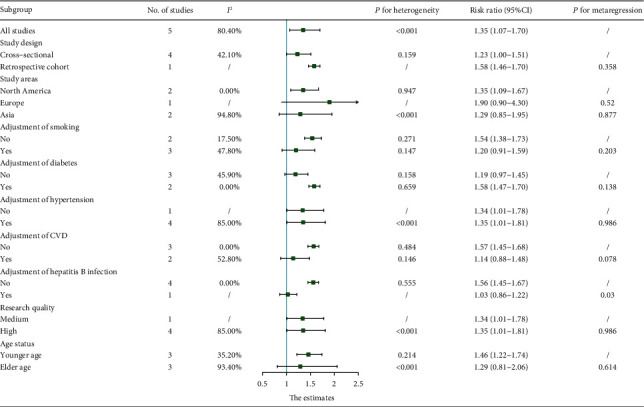
The results of the subgroup analysis and metaregression.

Figure 4 lists the details of the subgroup analysis and metaregression. The RR values of all subgroups were >1.00. According to the study design, whether to adjust smoking, diabetes, CVD, and hepatitis B infection grouping, the heterogeneity decreased significantly. But metaregression found that the differences between the subgroups were statistically significant only in the subgroup of hepatitis B infection.

#### 3.2.4. Publication Bias

According to the results of Egger's (*P* = 0.509) and Begg's test (*P* = 0.806), we found no obvious publication bias.

## 4. Discussion

There is no unified conclusion on whether PD and AMD are related, and the focus of each research result is different. Klein et al. [18] believed that the history of PD is related to the incidence of early AMD; Karesvuo et al. [19] confirmed that male alveolar bone loss is independently associated with AMD; Wagley et al. [20] found that PD is independently associated with AMD under 60 years of age or younger; Shin et al. [21] believe that in 62-year-old or younger people, only severe PD is independently related to AMD; cohort studies by Sun et al. [22] indicate that PD patients are more likely to develop AMD. And there is no relevant meta-analysis, so we think it is necessary to discuss whether PD and AMD are related in general. The meta-analysis results of the existing epidemiological evidence show that PD is associated with AMD, and the risk of AMD in PD patients is 1.35 times that of non-PD patients (95% CI: 1.07–1.70, *P* = 0.011). *I*^2^ = 80.4%, *P* for heterogeneity < 0.001, and it can be considered obvious heterogeneity. Through subgroup analysis, we found that the type of study, whether to adjust smoking, diabetes, CVD, and hepatitis B infection reduced heterogeneity. Combined with the metaregression results, whether to adjust for hepatitis B infection may be the source of heterogeneity. Some studies have shown that hepatitis B infection is also a risk factor for AMD [32], and only a study by Shin et al. [21] adjusted this confounding factor; smoking, hypertension, and CVD are common risk factors for PD and AMD [6, 33]. The results of the subgroup analysis show that the above three factors may cooperate with PD to increase the risk of AMD; diabetes is also a common risk factor for PD and AMD [11, 34], but our subgroup analysis results show that after adjusting for the confounding factor of diabetes, the correlation between PD and AMD was higher. We considered that there may be fewer studies included, and the results of Shin et al. may be the source of heterogeneity, but their study did not control the factor of diabetes, thus affecting the results of the diabetes subgroup. Subgroup analysis results according to the study quality show that the effect value did not change significantly, indicating that the overall research results are robust. Age is also a common and main risk factor for PD and AMD, and AMD mostly occurs in people over 50 years old. If the proportion of participants under 50 years old is too large, the prevalence of AMD may be underestimated in the original studies and then impact the accuracy of our results. There were 3 studies in the existing evidence that provided the effect sizes and 95% CIs of the younger and elder age groups. After subgroup analysis and metaregression, we found that the correlation between PD and AMD in the younger group (RR = 1.46, 95% CI: 1.22-1.74, *I*^2^ = 35.2%) was stronger than the elder group (RR = 1.29, 95% CI: 0.81-2.16, *I*^2^ = 93.4%). We believed that this may be due to the following reasons: First, we thought that PD may have a closer relationship with the early stages of AMD. Second, although the age mix factor was adjusted, the effects of PD on AMD were diluted with other changes in the body as we age. Finally, we have only three included studies grouped according to the age group, impacting the reliability of our final results. However, the result of metaregression showed that the difference between the younger and elder age groups was not statistically significant, indicating that although the included studies included participants under the age of 50, we may have underestimated the prevalence of AMD, but it did not affect the final results. But due to the lack of original data, we could not adjust the age in the metaregression of each subgroup; these factors affected the reliability of metaregression in our study. After the sensitivity analysis excludes one study at a time, the meta-analysis results have not changed significantly. Egger's test and Begg's test did not find obvious bias.

In recent years, many studies have shown that periodontal infection may be a risk factor for cardiovascular disease, diabetes, pregnancy complications, respiratory infections, rheumatoid arthritis, and so on [8–12]. Periodontal bacteria and their products enter the systemic circulation, activate immune cells in other body parts to release proinflammatory cytokines, and form a chronic inflammatory state, which in turn leads to the development of some diseases, such as atherosclerosis [35] and premature delivery [36]. Studies have found oral pathogens in human atherosclerotic plaques [37], and Kalayoglu et al. [38] have found oral pathogens—Chlamydia pneumoniae—in human choroidal neovascular membranes excised from patients with wet AMD. We speculated that PD may play a role in the development of AMD through a mechanism similar to the above. The pathogenesis of AMD is unclear, but the theory of inflammation and immunology has received increasing attention from researchers [13–15]. Aging causes a decrease in the ability of retinal pigmented epithelium (RPE) cells to clear metabolic products, aggravating oxidative damage to the retina, and synergistic effects of metabolic, functional, genetic, and environmental risk factors, resulting in the accumulation of retinal toxic elements. In response to the increase in tissue damage, various immune pathways are inappropriately activated, including the classical and alternative complement pathways, the inflammasome, and Toll-like receptor (TLR) signaling. Eventually, the continuous activation of these proinflammatory and injury pathways leads to the development of advanced AMD and irreversible loss of vision [39]. Therefore, we have reason to believe that inflammation plays an important role in the relationship between PD and AMD. Periodontal infection may activate immune pathways on the retina through systemic circulation to promote the development of AMD.

Our research has the following advantages. First, this study is the first meta-analysis about whether PD and AMD are related. We retrieved and collected all published studies that met the inclusion criteria, and no publication bias was detected. Second, PD and AMD have some common risk factors; all the studies we included provided the effect sizes after adjusting for related confounding factors. In addition, the quality of the included studies were all medium-/high-quality studies, which made our results more reliable. Finally, this study laid the foundation for further exploration of the relationship between PD and AMD and provided a certain theoretical basis for AMD's mechanism exploration and clinical prevention and treatment.

Our research also has some limitations. First, most of the included studies are cross-sectional studies, which cannot explain the causal relationship between PD and AMD. Second, although most studies were based on scientific examination methods to diagnose PD and AMD, the diagnostic criteria were different, and some diagnoses were based on patient self-reports, which increased the heterogeneity of our research and reduced credibility. Third, we observed significant heterogeneity in the study. Although the subgroup analysis was used to speculate on the source of the heterogeneity, it still had an impact on the reliability of the research results. Fourth, Egger's test and Begg's test results did not find obvious evidence, but because of the too few studies included, it is difficult to determine whether there is publication bias. Fifth, the adjustment of confounding factors was not perfect. Although each study had carried out meticulous research design and tried to control the confounding factors, there were still unmeasured or unknown confounding factors, which may introduce deviations. And the confounding factors adjusted between different studies were different, which increased the heterogeneity of the study and affected the reliability of the final result. Finally, due to the limited number of studies, we only analyzed the relationship between PD and AMD in general and could not further explore the impact of PD on AMD patients at different stages or at different ages and the association between PD at different severity levels and AMD.

With the development of the economy and society, the average lifespan of the population has been prolonged, resulting in population aging. It is the responsibility of oral professionals to reduce the incidence of oral infections and possible systemic diseases caused by PD through early treatment. AMD can cause severe irreversible vision loss in the elderly, which is one of the important causes of blindness. At present, most clinical cases have no effective treatment, which seriously affects the lives of patients. We hope that our research will improve the oral health awareness, help in understanding the importance of maintaining periodontal health, and at the same time lay the foundation for AMD's mechanism exploration and prevention, reduce the risk of AMD, and improve the quality of life in the general population, especially the geriatric population.

## 5. Conclusion

In summary, our meta-analysis of 5 studies showed that PD is associated with AMD, and the risk of AMD in patients with PD is increased by 35%, but the current evidence is insufficient to confirm the causal relationship between PD and AMD. Researchers need to perform high-quality, large-scale, multi-centre prospective cohort studies and adjust the confounding factors in considerable detail to explore whether these two important diseases are related to each other and the mechanism of their connection.

## Figures and Tables

**Table 1:** Characteristics and quality assessment of the included studies.

First author, published year	Sample study period	Study design	Follow-up period	Age (yrs)	AMD patients/participants	PD ascertainment	AMD ascertainment	AMD/age status	Risk estimates (95% CI)
Klein, 2008	MESA; USA 2002-2004	Cross-sectional	2	45-85	265/5887	Detailed questionnaire	Ocular fundus photographs	Early Late All^#^	OR = 1.32 (0.98-1.79) 1.49 (0.63-3.50) 1.34 (1.01-1.78)
Karesvuo, 2013	NPHS; Finland 2000-2001	Cross-sectional	1	≥30	54/1751	Alveolar bone loss	Clinical diagnosis	—	OR = 1.90 (0.90-4.30)
Wagley, 2015	US NHAHES III; US 1988-1994	Cross-sectional	6	≥40	940/8208	>10% of sites with >3 mm of CAL	Ocular fundus photographs	Age ≤ 60 Age > 60 All^&^	OR = 1.96 (1.22-3.14) 1.32 (0.93-1.90) 1.36 (0.98-1.90)
Shin, 2017	KNHANES; Korea 2008-2010 and 2012	Cross-sectional	2	≥40	732/13072	CP index scores 3 and 4	Ocular fundus photographs	Age ≤ 62 Age > 62 All^&^	OR = 1.21 (0.90-1.63) 0.91 (0.74-1.12) 1.03 (0.86-1.22)
Sun, 2019	TNHIRD; China 2000-2012	Retrospective cohort	13	≥50	3014/83322	ICD-9-CM	ICD-9-CM	Age ≤ 64 Age ≥ 65 All^&^	HR = 1.48 (1.34-1.64) 1.76 (1.57-1.97) 1.58 (1.46-1.70)

MESA: Multi-Ethnic Study of Atherosclerosis; NPHS: National Population Health Survey 2000; NHAHES: National Health and Nutrition Examination Survey; KNHANES: Korea National Health and Nutrition Examination Survey; TNHIRD: Taiwan National Health Insurance Research Database; ICD: International Classification of Diseases. ^#^All data was calculated by pooling early AMD *P* and late AMD *P* using a fixed-effects model for the final analysis; ^&^all data was calculated by pooling different age status *P* using a fixed-effects model for the final analysis.

**Table 2:** Adjusted variables in studies included in the meta-analysis.

Author (year)	Variables of adjustment
Klein (2008)	Age, gender, race/ethnicity, and study site.
Karesvuo (2013)	Age, smoking, diabetes, carriage of salivary pathogens, and hypertension.
Wagley (2015)	Age, gender, race/ethnicity, education, poverty income ratio, smoking, hypertension, BMI, CRP, and CVD.
Shin (2017)	Age, gender, education, house income, smoking, hypertension, CVD, anemia, hepatitis B infection, serum HDL level, BMI, serum ferritin level, and white blood cell count.
Sun (2019)	Age, gender, hypertension, diabetes, hyperlipidemia, asthma/COPD, CLD, and CKD.

Abbreviations: BMI: Body Mass Index; CRP: C-reactive protein; CVD: cardiovascular disease; HDL: high-density lipoprotein; COPD: chronic obstructive pulmonary disease; CLD: chronic liver disease and cirrhosis; CKD: chronic kidney disease.

**Table 3:** Agency for Healthcare Research and Quality: cross-sectional studies.

Study ID	Source of information^a^	Exclusion criteria^b^	Time period^c^	Continuous or not^d^	Subjective factors^e^	Quality assessment^f^	Explain exclusion^g^	Controlling confounding factors^h^	Handling missing data^i^	Completeness of data collection^j^	Follow-up data^k^	Score
Klein 2008	Yes	Yes	Yes	Yes	Yes	No	No	Yes	No	No	No	6
Karesvuo 2013	Yes	Yes	Yes	Yes	Yes	Yes	No	Yes	No	Yes	No	8
Wagley 2015	Yes	Yes	Yes	Yes	Yes	Yes	Yes	Yes	No	No	No	8
Shin 2017	Yes	Yes	Yes	Yes	Yes	Yes	Yes	Yes	No	No	No	8

a: define the source of information (survey, record review); b: list inclusion criteria for exposed and unexposed subjects (cases and controls) or refer to previous publications; c: indicate time period used for identifying patients; d: indicate whether or not subjects were consecutive if not population-based; e: indicate if evaluators of subjective components of the study were masked to other aspects of the status of the participants; f: describe any assessments undertaken for quality assurance purposes (e.g., test/retest of primary outcome measurements); g: explain any patient exclusions from analysis; h: describe how confounding factors were assessed and/or controlled; i: if applicable, explain how missing data were handled in the analysis; j: summarize patient response rates and completeness of data collection; k: clarify what follow-up was expected and the percentage of patients for which incomplete data or follow-up was obtained.

**Table 4:** Newcastle-Ottawa Quality Assessment Scale: cohort studies.

Study ID	Selection				Comparability^e^		Exposure			Total scores
	Representativeness^a^	Selection^b^	Ascertainment^c^	Demonstration^d^	Important factor^f^	Additional factor^g^	Assessment^h^	Follow-up		
								Length^i^	Adequacy^j^	
Sun 2019	Representative^★^	Same source^★^	Secure record^★^	Yes^★^	Yes^★^	Yes^★^	Record linkage^★^	Yes^★^	No description	8^★^

a: representativeness of the exposed cohort; b: selection of the nonexposed cohort; c: ascertainment of exposure; d: demonstration that outcome of interest was not present at the start of study; e: comparability of cohorts on the basis of the design or analysis; f: study controls for selecting the most important factor; g: study controls for any additional factor; h: assessment of outcome; i: was follow-up long enough for outcomes to occur; j: adequacy of follow-up of cohorts. ★: earns a star.

## Supplementary Materials

Supplementary Materials Appendix 1: electronic search criteria.
