# Supplementary material for: Periodontal Disease and Age-Related Macular Degeneration: A Meta-Analysis of 112,240 Participants
Source: Biomed Res Int. 2020 Sep 28;2020:4753645. doi: 10.1155/2020/4753645 (PMC7542535; doi:10.1155/2020/4753645)
Supplement: Supplementary Materials — Appendix 1: electronic search criteria. [file 4753645.f1.pdf]

Appendix 1:Electronic Search Criteria

PubMed

#1 "Periodontal Diseases"[Mesh]

#2 "disease periodontal" OR "diseases periodontal" OR "periodontal disease" OR "Parodontosis" OR "Parodontoses" OR "pyorrhea alveolaris"

#3 "Furcation Defects"[MeSH Terms] OR "Gingivitis"[MeSH Terms] OR "peri-implantitis"[MeSH Terms] OR "Periodontitis"[MeSH Terms] OR "Alveolar Bone Loss"[MeSH Terms] OR "Periodontal Attachment Loss"[MeSH Terms]

#4 "defect furcation" OR "defects furcation" OR "Furcation Defect" OR "Gingivitides" OR "Peri Implantitis" OR "Peri-Implantitides" OR "Periimplantitis" OR "Periimplantitides" OR "Periodontitides" OR "Pericementitis" OR "Alveolar Bone Losses" OR "Alveolar Process Atrophy" OR "Alveolar Process Atrophies" OR "Alveolar Resorption" OR "resorption alveolar" OR "bone loss periodontal" OR "Periodontal Bone Losses" OR "Periodontal Bone Loss" OR "Periodontal Resorption" OR "resorption periodontal" OR "Alveolar Bone Atrophy" OR "bone loss alveolar" OR "attachment loss periodontal" OR "loss periodontal attachment"

#5 #1 OR #2 OR #3 OR #4

#6 "Macular Degeneration"[Mesh] OR "Retinal Drusen"[Mesh] OR "Optic Disk Drusen"[Mesh] OR "Geographic Atrophy"[Mesh] OR "Retinal Detachment"[Mesh] OR "Choroidal Neovascularization"[Mesh]

#7 "Degeneration, Macular" OR "Macular Degenerations" OR "Maculopathy" OR Maculopathies OR "Macular Dystrophy" OR "Dystrophy, Macular" OR "Macular Dystrophies" OR "Age-Related Macular Degeneration" OR "Age Related Macular Degeneration" OR "Age-Related Macular Degenerations" OR "Macular Degeneration, Age-Related" OR "Macular Degeneration, Age Related" OR "Maculopathies, Age-Related" OR "Maculopathy, Age-Related" OR "Maculopathy, Age Related" OR "Age-Related Maculopathies" OR "Age Related Maculopathies" OR "Age-Related Maculopathy" OR “AMD”

#8 "Drusen, Retinal" OR "Optic Disc Drusen" OR "Drusen, Optic Nerve" OR "Optic Nerve Drusen" OR "Drusen, Optic Disc" OR "Optic Disc Drusens" OR "Drusen, Optic Disk" OR "Optic Disk Drusens" OR "Drusen"

#9 "Atrophies, Geographic" OR "Atrophy, Geographic" OR "Geographic Atrophies" OR "Dry Macular Degeneration" OR "Degeneration, Dry Macular" OR "Degenerations, Dry Macular" OR "Dry Macular Degenerations" OR "Macular Degeneration, Dry" OR "Macular Degenerations, Dry"

#10 "Detachment, Retinal" OR "Detachments, Retinal" OR "Retinal Detachments" OR "Retinal

Pigment Epithelial Detachment"

#11 "Neovascularization, Choroid" OR "Neovascularization, Choroidal" OR "Choroidal Neovascularizations" OR "Choroid Neovascularization" OR "Choroid Neovascularizations"

#12 "Polypoidal choroidal vasculopathy"

#13 #6 OR #7 OR #8 OR #9 OR #10 OR #11 OR #12

#14 #5 AND #13

Embase

#1 'periodontal disease'/exp

#2 'gingivitis'/exp OR 'periimplantitis'/exp OR 'alveolar bone loss'/exp OR 'periodontitis'/exp

#3 'furcation defects' OR 'defect furcation' OR 'defects furcation' OR 'furcation defect' OR gingivitides OR 'peri implantitis' OR 'peri implantitides' OR periimplantitides OR periodontitides OR pericementitis OR 'alveolar bone losses' OR 'alveolar process atrophy' OR 'alveolar process atrophies' OR 'alveolar resorption' OR 'resorption alveolar' OR 'bone loss periodontal' OR 'periodontal bone losses' OR 'periodontal bone loss' OR 'periodontal resorption' OR 'resorption periodontal' OR 'alveolar bone atrophy' OR 'bone loss alveolar' OR 'attachment loss periodontal' OR 'loss periodontal attachment' OR 'periodontal attachment loss'

#4 'periodontal diseases' OR 'disease periodontal' OR parodontosis OR parodontoses OR 'pyorrhea alveolaris'

#5 'macular degeneration'/exp OR 'drusen'/exp OR 'geographic atrophy'/exp OR 'retina detachment'/exp OR 'subretinal neovascularization'/exp OR 'Polypoidal choroidal vasculopathy'/exp

#6 'degeneration, macular' OR 'macular degenerations' OR 'maculopathy' OR maculopathies OR 'macular dystrophy' OR 'dystrophy, macular' OR 'macular dystrophies' OR 'age-related macular degeneration' OR 'age related macular degeneration' OR 'age-related macular degenerations' OR 'macular degeneration, age-related' OR 'macular degeneration, age related' OR 'maculopathies, age-related' OR 'maculopathy, age-related' OR 'maculopathy, age related' OR 'age-related maculopathies' OR 'age related maculopathies' OR 'age-related maculopathy' OR 'amd' OR 'atrophies, geographic' OR 'atrophy, geographic' OR 'geographic atrophies' OR 'dry macular degeneration' OR 'degeneration, dry macular' OR 'degenerations, dry macular' OR 'dry macular degenerations' OR 'macular degeneration, dry' OR 'macular degenerations, dry' OR 'retinal detachment' OR 'detachment, retinal' OR 'detachments, retinal' OR 'retinal detachments' OR 'retinal pigment epithelial detachment' OR 'neovascularization, choroid' OR 'neovascularization, choroidal' OR 'choroidal neovascularizations' OR 'choroid neovascularization' OR 'choroid neovascularizations'

#7 #1 OR #2 OR #3 OR #4

#8 #5 OR #6

#9 #7 AND #8

Cochrane Library

#1 MeSH descriptor: [Periodontal Diseases] explode all trees

#2 MeSH descriptor: [Furcation Defects] explode all trees

#3 MeSH descriptor: [Gingivitis] explode all trees

#4 MeSH descriptor: [Peri-Implantitis] explode all trees

#5 MeSH descriptor: [Periodontitis] explode all trees

#6 MeSH descriptor: [Periodontal Attachment Loss] explode all trees

#7 ((Disease, Periodontal) OR (Diseases, Periodontal) OR (Periodontal Disease) OR (Parodontosis) OR (Parodontoses) OR (Pyorrhea Alveolaris) OR (defect furcation) OR (defects furcation) OR (Furcation Defect) OR (Gingivitides) OR (Peri Implantitis) OR (Peri-Implantitides) OR (Periimplantitis) OR (Periimplantitides) OR (Periodontitides) OR (Pericementitis) OR (Alveolar Bone Losses) OR (Alveolar Process Atrophy) OR (Alveolar Process Atrophies) OR (Alveolar Resorption) OR (resorption alveolar) OR (bone loss periodontal) OR (Periodontal Bone Losses) OR (Periodontal Bone Loss) OR (Periodontal Resorption) OR (resorption periodontal) OR (Alveolar Bone Atrophy) OR (bone loss alveolar) OR (attachment loss periodontal) OR (loss periodontal attachment)):ti,ab,kw (Word variations have been searched)

#8 #1 OR #2 OR #3 OR #4 OR #5 OR #6 OR #7

#9 MeSH descriptor: [Macular Degeneration] explode all trees

#10 MeSH descriptor: [Retinal Drusen] explode all trees

#11 MeSH descriptor: [Optic Disk Drusen] explode all trees

#12 MeSH descriptor: [Geographic Atrophy] explode all trees

#13 MeSH descriptor: [Retinal Detachment] explode all trees

#14 MeSH descriptor: [Choroidal Neovascularization] explode all trees

#15 (Degeneration, Macular) OR (Macular Degenerations) OR (Maculopathy) OR Maculopathies OR (Macular Dystrophy) OR (Dystrophy, Macular) OR (Macular Dystrophies) OR (Age-Related Macular Degeneration) OR (Age Related Macular Degeneration) OR (Age-Related Macular Degenerations) OR (Macular Degeneration, Age-Related) OR (Macular Degeneration, Age Related) OR (Maculopathies, Age-Related) OR (Maculopathy, Age-Related) OR (Maculopathy, Age Related) OR (Age-Related Maculopathies) OR (Age Related Maculopathies) OR (Age-Related Maculopathy) OR (AMD)

#16 (Drusen, Retinal) OR (Optic Disc Drusen) OR (Drusen, Optic Nerve) OR (Optic Nerve Drusen) OR (Drusen, Optic Disc) OR (Optic Disc Drusens) OR (Drusen, Optic Disk) OR (Optic Disk Drusens) OR (Drusen)

#17 (Atrophies, Geographic) OR (Atrophy, Geographic) OR (Geographic Atrophies) OR (Dry Macular Degeneration) OR (Degeneration, Dry Macular) OR (Degenerations, Dry Macular) OR (Dry Macular Degenerations) OR (Macular Degeneration, Dry) OR (Macular Degenerations, Dry)

#18 (Detachment, Retinal) OR (Detachments, Retinal) OR (Retinal Detachments) OR (Retinal Pigment Epithelial Detachment)

#19 (Neovascularization, Choroid) OR (Neovascularization, Choroidal) OR (Choroidal Neovascularizations) OR (Choroid Neovascularization) OR (Choroid Neovascularizations)

#20 (Polypoidal choroidal vasculopathy)

#21 #9 OR #10 OR #11 OR #12 OR #13 OR #14 OR #15 OR #16 OR #17 OR #18 OR #19 OR #20

#22 #8 AND #21

Web of Science

#1 TS=('periodontal diseases')

#2 TS=('disease periodontal' OR 'diseases periodontal' OR 'periodontal disease' OR 'Parodontosis' OR 'Parodontoses' OR 'pyorrhea alveolaris')

#3 TS=('furcation defects' OR 'gingivitis' OR 'peri-implantitis' OR 'periodontitis' OR 'alveolar bone loss' OR 'periodontal attachment loss')

#4 TS=('defect furcation' OR 'defects furcation' OR 'Furcation Defect' OR 'Gingivitides' OR 'Peri Implantitis' OR 'Peri-Implantitides' OR 'Periimplantitis' OR 'Periimplantitides' OR 'Periodontitides' OR 'Pericementitis' OR 'Alveolar Bone Losses' OR 'Alveolar Process Atrophy' OR 'Alveolar Process Atrophies' OR 'Alveolar Resorption' OR 'resorption alveolar' OR 'bone loss periodontal' OR 'Periodontal Bone Losses' OR 'Periodontal Bone Loss' OR 'Periodontal Resorption' OR 'resorption periodontal' OR 'Alveolar Bone Atrophy' OR 'bone loss alveolar' OR 'attachment loss periodontal' OR 'loss periodontal attachment')

#5 #1 OR #2 OR #3 OR #4

#6 TS=('macular degeneration' OR 'retinal drusen' OR 'optic disk drusen' OR 'geographic atrophy' OR 'retinal detachment' OR 'choroidal neovascularization')

#7 TS=('Degeneration, Macular' OR 'Macular Degenerations' OR 'Maculopathy' OR Maculopathies OR 'Macular Dystrophy' OR 'Dystrophy, Macular' OR 'Macular Dystrophies' OR

'Age-Related Macular Degeneration' OR 'Age Related Macular Degeneration' OR 'Age-Related Macular Degenerations' OR 'Macular Degeneration, Age-Related' OR 'Macular Degeneration, Age Related' OR 'Maculopathies, Age-Related' OR 'Maculopathy, Age-Related' OR 'Maculopathy, Age Related' OR 'Age-Related Maculopathies' OR 'Age Related Maculopathies' OR 'Age-Related Maculopathy' OR ' AMD')

#8 TS=('Drusen, Retinal' OR 'Optic Disc Drusen' OR 'Drusen, Optic Nerve' OR 'Optic Nerve Drusen' OR 'Drusen, Optic Disc' OR 'Optic Disc Drusens' OR 'Drusen, Optic Disk' OR 'Optic Disk Drusens' OR 'Drusen')

#9 TS=('Atrophies, Geographic' OR 'Atrophy, Geographic' OR 'Geographic Atrophies' OR 'Dry Macular Degeneration' OR 'Degeneration, Dry Macular' OR 'Degenerations, Dry Macular' OR 'Dry Macular Degenerations' OR 'Macular Degeneration, Dry' OR 'Macular Degenerations, Dry')

#10 TS=('Detachment, Retinal' OR 'Detachments, Retinal' OR 'Retinal Detachments' OR 'Retinal Pigment Epithelial Detachment')

#11 TS=('Neovascularization, Choroid' OR 'Neovascularization, Choroidal' OR 'Choroidal Neovascularizations' OR 'Choroid Neovascularization' OR 'Choroid Neovascularizations')

#12 TS=('Polypoidal choroidal vasculopathy')

#13 #6 OR #7 OR #8 OR #9 OR #10 OR #11 OR #12

#14 #5 AND #13
